# SARS-CoV-2-Induced Multisystem Inflammatory Syndrome in a Young Adult: Case Report

**DOI:** 10.1007/s42399-021-00998-x

**Published:** 2021-06-20

**Authors:** Haldun Bulut, Alexandra H. E. Herbers, Ilse M. G. Hageman, Paetrick M. Netten, Hendrik J. M. de Jonge, Robert Joustra, Frank L. van de Veerdonk, Cornelis P. C. de Jager

**Affiliations:** 1 Department of Internal Medicine, Radboud University Medical Center, Nijmegen, The Netherlands; 2 Department of Internal Medicine, Jeroen Bosch Hospital, ‘s-Hertogenbosch, The Netherlands; 3 Department of Gastroenterology and Hepatology, Jeroen Bosch Hospital, ‘s-Hertogenbosch, The Netherlands; 4 Department of Cardiology, Jeroen Bosch Hospital, ‘s-Hertogenbosch, The Netherlands; 5 Department of Intensive Care Medicine, Jeroen Bosch Hospital, ‘s-Hertogenbosch, The Netherlands

**Keywords:** COVID-19, Multisystem inflammatory syndrome, Heart failure, Immunoglobulins, Glucocorticoids

## Abstract

We describe a case of a previous healthy 20-year-old male athlete who presented with an atypical clinical profile with multiorgan involvement within five weeks after confirmed SARS-CoV-2 infection, suggestive for multisystem inflammatory syndrome (MIS); MIS is a rare, potentially life-threatening complication associated with SARS-CoV-2. MIS shares similar clinical features compatible with several overlapping lifethreatening hyperinflammatory syndromes, such as incomplete Kawasaki Disease (KD) and toxic shock syndrome (TSS) associated to a cytokine storm suggestive of a macrophage activation syndrome (MAS) without fulfilling the criteria for hemophagocytic lymphohistiocytosis (HLH), that may create a great challenge to distinguish between them. MIS should promptly be considered and treated, as uncontrolled MIS has a high mortality.

In MIS cardiac involvement, heart failure may present as an additional problem, especially because volume loading is advised in accordance with proposed therapy. Carefully monitoring of the respiratory and cardiac status in response of resuscitation is therefore warranted.

## Introduction

SARS-CoV-2 has globally resulted in more than twenty million of infected cases and deaths [2]. Recent data suggest that in children infected with SARS-CoV-2, the course of the disease is often mild or even sub-clinical [3]. Young adult patients with a new clinical profile manifesting as a hyperinflammatory syndrome, i.e., severe multiorgan involvement, rapidly evolving into acute multiorgan failure, several weeks after infected with SARS-CoV-2, are globally reported [4–15]. They present with a similar pattern of health complaints and clinical course, consisting of persistent high grade of fever (99%), gastrointestinal (94%) and neurological (68%) symptoms, elevated cardiac (75%) and inflammatory biomarkers (99%), and cytopenia (76%). A substantial number of them eventually develop refractory shock requiring vasopressive therapy and invasive mechanical ventilation. This clinical profile  was reminiscent to TSS, KD, and HLH ([1], Table 3 in reference [17]). The Centre for Disease Control and Prevention has named this multisystem inflammatory syndrome (MIS).

MIS is a rare but increasingly recognized complication of SARS-CoV-2 infection, usually presenting 2 up to 6 weeks after the onset of the COVID-19 infection symptoms [5] and affecting mainly children. Any patient with suspected MIS should be evaluated for infectious and non-infectious etiologies. Early recognition of MIS is important because it is associated with high mortality if left untreated. However, when MIS is suspected, HLH and other overlapping hyperinflammatory syndromes should be in the differential diagnosis. In the present report, we discuss the red flags, diagnostic, and therapeutic challenges in MIS triggered by SARS-CoV-2.

## Case Description

A previously healthy 20-year-old Caucasian male patient with no medical history was admitted to our hospital with high fever up to 40 °C since 5 days, abdominal pain and diarrhea. There was no history of weight loss or night sweats. Family history of malignancies and inflammatory bowel diseases was negative. Several weeks before presentation, he was infected with SARS-CoV-2 and completely recovered from the infection within 2 weeks.

**Fig. 1:**
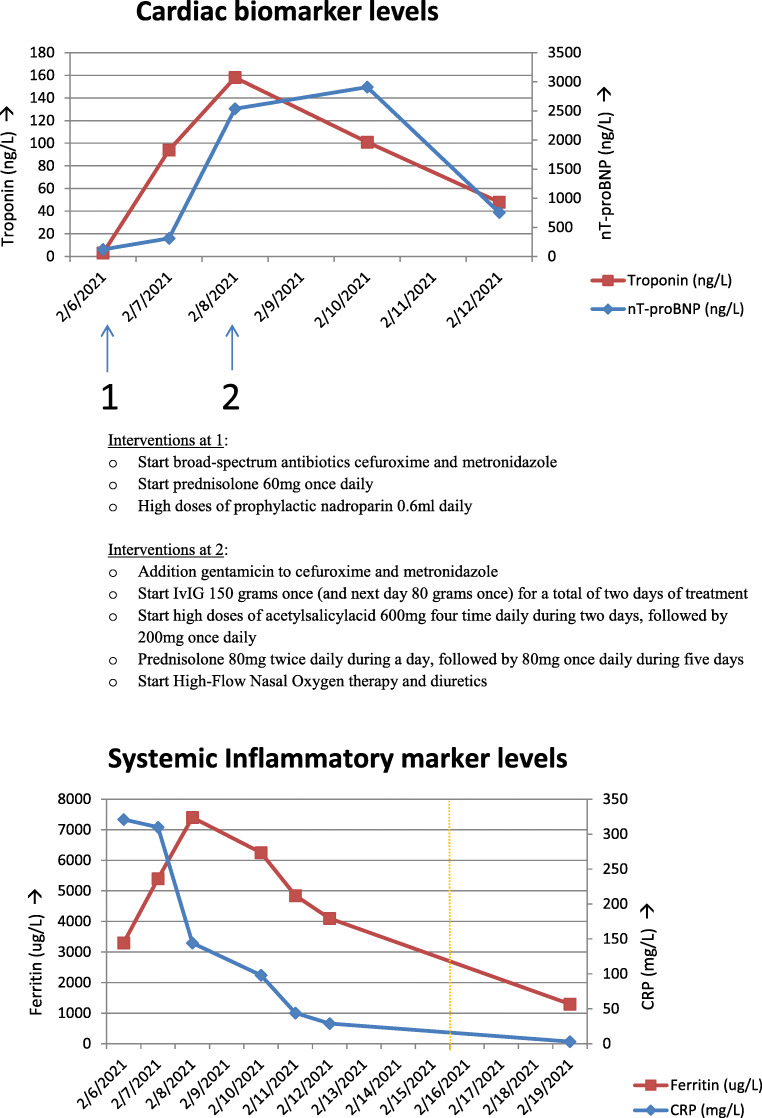
**A** Timeline of cardiac biomarker levels in response to interventions throughout hospital stay. Interventions at 1 are as follows: start broad-spectrum antibiotics cefuroxime and metronidazole, start prednisolone 60 mg once daily, and high doses of prophylactic nadroparin 0.6 mL daily. Interventions at 2 are as follows: addition gentamicin to cefuroxime and metronidazole; start IVIG 150 g once (and next day 80 g once) for a total of 2 days of treatment; start high doses of acetylsalicylacid 600 mg four times daily during 2 days, followed by 200 mg once daily; prednisolone 80 mg twice daily during a day, followed by 80 mg once daily during 5 days; and start high-flow nasal oxygen therapy and diuretics. **B** Timeline of the inflammatory marker levels in response to the described interventions above

We observed an acute ill-painfull-looking young man. Physical examination revealed a blood pressure of 95/40 mmHg with sinustachycardia of 110 beats per minute, a respiratory rate of twenty-four per minute, pulse oximetry of 97% without supplied oxygen, and temperature of 39.8 °C. Remaining clinical examination displayed no meningism signs, no cardiac murmurs, no skin abnormalities, and a soft abdomen with pressure pain in the lower right part at the McBurney’s point. Laboratory testing revealed high levels of inflammatory biomarkers, including ferritin, elevated lactate dehydrogenase and liver enzymes, and normocytic anemia without signs of hemolysis (see Table 1 and Fig. 1). The contrast enhanced computed tomographic scan (CT) of the thorax and abdomen revealed splenomegaly and thickening of the terminal ileum in a long traject of at least 20 cm. No pulmonary infiltrates or signs of malignancy were detected. The SARS-CoV-2 PCR on a smear from the throat and nose and the SARS-CoV-2 IgG antibody titre (94.7 AU/mL) were positive. Serology was negative for human immunodeficiency virus, cytomegalovirus, and Epstein-Barr virus. Blood, urinary, and feces cultures were negative. An infectious terminal ileitis was suspected for which patient received broad-spectrum antibiotic therapy (cefuroxime and metronidazole). Despite this therapy, the patient’s clinical course showed no improvement in a couple of hours. A worsening with additional neurological symptoms followed. The patient did not fulfill the criteria for HLH, MAS or KD. MIS was suspected, and prednisolone 60 mg once daily was started shortly after admission (see Table 1 in reference [17]).

**Table 1:** Patient characteristics during and after hospitalization

	Day 0	Day 2	Day 6	1 week after discharge	4 weeks after discharge	Reference values
Hemoglobin (g/dL)	7.7	7.2	8.0	8.6	9.1	12–18 g/dL
White cell count (10^9^ cells/L)	6.9	6.5	13.6	5.9	5.8	4–11 × 10^9^ cells/L
Lymphocyte count (10^9^ cells/L)	0.3	0.6			2.8	1.0–3.5 × 10^9^ cells/L
Platelet count (10^9^ cells/L)	133	113	368	596	248	150–400 × 10^9^ cells/L
Lactate dehydrogenase (U/L)	418	379	335		197	<250 U/L
Ferritin (μg/L)	3300	7400	4100	1300	190	20–300 μg/L
CRP (mg/L)	310	144	29	3	< 3	0–8 mg/L
Procalcitonin (ng/L)	8.1	8.0	0.82			0–0.5 ng/L
D-dimer (mg/L)	7.44	4.37				2.0–4.0 mg/L
Fibrinogen (mg/L)	1050	1480				1600–3200 mg/L
Hs Troponin I (ng/L)	3	158	48		6	0–47 (ng/L)
nT-proBNP (ng/L)	120	2538	756		< 35	0–125 (ng/L)

**Fig. 2:**
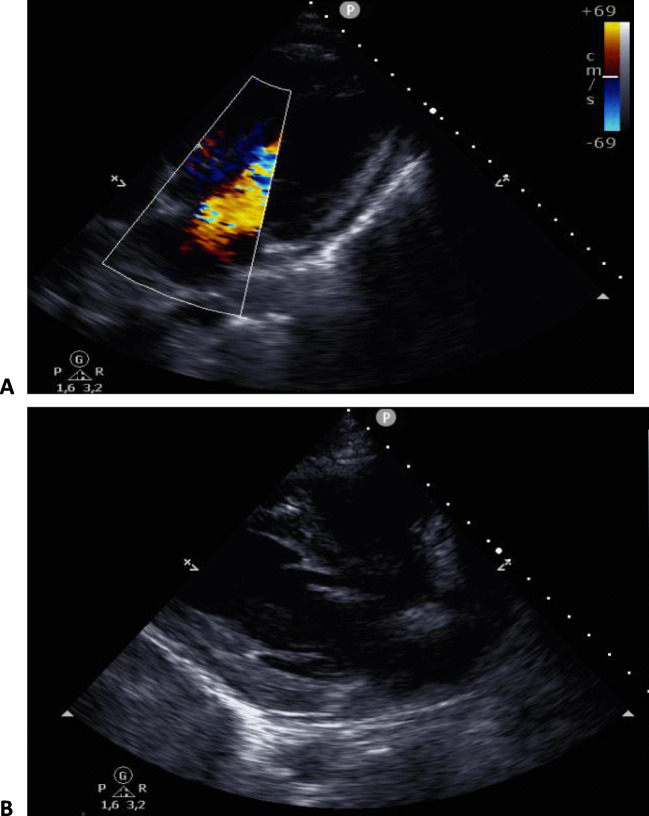
Images of echocardiogram at hospitalization day 2; suspicion on cardiac involvement as part of MIS. The panels below demonstrate depressed systolic left ventricular function, slight tricuspidal valve insufficiency, aotic valve with 3 leaflets without pericardial effusion, and a dilated vena cava inferior

Thirty hours later, the patient’s condition deteriorated rapidly with additional chest pain and hemodynamic instability, i.e., resuscitation refractory hypotension (80/39 mmHg) with sinustachycardia of 115 beats per minute. Laboratory tests showed increased ferritin levels, pancytopenia and elevated inflammatory, and cardiac biomarkers. Electrocardiogram revealed no abnormalities. Broadening of the antibiotic regimen with gentamicin, intravenous immune globulins (IVIG) during 2 days (2 g/kg, 150 g and 1 g/kg, and 80 g, respectively), and aspirin (200 mg daily) were initiated. After fluid resuscitation accompanying immune globulin treatment, respiratory failure occurred. The CT-angiography of the chest did not show evidence of pulmonary embolism or infiltrates. The echocardiogram revealed a moderately reduced systolic left ventricular function with estimated ejection fraction of below 40%, with global hypokinesia without regional wall motion abnormalities related to a circulation area of a specific coronary artery (see Fig. 2). Cardiac involvement as part of MIS was hypothesized. High-flow oxygen therapy was started, after which his respiratory condition recovered well within 24 h. The first follow-up echocardiography, performed at the 10th day of hospitalization, showed significantly improvement of left ventricular systolic function with estimated ejection fraction of 50%.

Forty-eight hours after completing the therapy with IVIG and the started higher doses of prednisolone and aspirin, the patient’s clinical condition and laboratory tests normalized gradually (see Table 1 and Fig. 1). The therapy with prednisone was continued in the following schedule: 80 mg twice daily during a day, followed by 80 mg once daily during 5 days, followed by 40 mg once daily from the date of discharge. The patient could be discharged on the eleventh day of hospitalization. The discharge medication consisted of prednisolone 40 mg once daily in phase-out schedule tapered over at least a week, a total of 22 days of treatment, aspirin 200 mg once daily with omeprazole to be continued for a total of 40 days. The first outpatient follow-up visit, seven days after discharge, showed further signs of health recovery, i.e., no recidive of fever and declining inflammatory parameters. The outpatient follow-up echocardiogram, six weeks after discharge, showed further recovery of the systolic left ventricular ejection fraction up to 60% without any heart valve defects. No remaining cardiac complaints were left.

## Discussion

The described MIS in this patient was characterized by a wide range of atypical symptoms. The patient presented to our hospital within a time period of  five weeks after being tested positive with the SARS-CoV-2. This similar interval between confirmed infection with SARS-CoV-2 and the onset of symptoms belonging to MIS is described in recent cases and made us hypothesize the patient would have MIS.

MIS shares many of the key clinical features with several overlapping hyperinflammatory syndromes, each with its own distinctive treatment, suggesting that MIS is part of a broad spectrum of diseases ([1], Table 3 in reference [17]). A recent positive SARS-CoV-2 serology test is the differentiating key factor of MIS from the overlapping syndromes. Because untreated MIS and other overlapping hyperinflammatory syndromes both are associated with a high mortality, it is important to also consider one of the overlapping hyperinflammatory syndromes in patients presenting with a condition of MIS or vice versa.

So far, mainly children with a subclinical infection with SARS-COV-2 that develop MIS have been described. Our patient was adult and initially did not have pulmonary symptoms, arterial hypoxemia, or radiographic distinctive features. Early echocardiogram revealed a depressed systolic left ventricular function without pericardial effusion (see Fig. 2). Pulmonary embolism and infectious pulmonary infiltrates were ruled out. The left ventricular function largely improved after high-flow oxygen and diuretic therapy. The observed positive effects of diuretics on the clinical course and systolic ventricular function suggest that iatrogenic fluid overload after resuscitation during 72 h may be the main underlying cause of the observed heart failure. However, the cardiac biomarkers and systolic ventricular function have not been completely recovered after the therapy; therefore, cardiac involvement as part of MIS is very likely. Resuscitation remains part of the proposed therapy in patients with severe MIS, but heart failure may be a relevant clinical problem. Therefore, monitoring of the cardiac status in response of the fluid therapy is warranted.

Cardiac manifestations in the light of MIS are seen in up to 80% of the cases, including arrhythmia, coronary artery involvement, and even aneurysms in addition to the abovementioned cardiac aspects [6]. The most severe cases may present cardiogenic shock requiring fluid resuscitation, vasopressive therapy, mechanical ventilation, and in exceptional cases even ECMO therapy (extracorporeal membrane oxygenation). Most of the affected patients (up to 78%) show full recovery of their left ventricular function, while up to 22% retail mild to moderate permanently decreased function after adequate therapy [7–10].

To date, there are no widely accepted guidelines yet for the optimal therapeutic approach to young adults with MIS. Treatment modalities have been extrapolated from suggested therapies in guidelines for the known overlapping syndromes. Given the clinical and pathophysiological similarities between MIS and incomplete KD and the effectiveness of IVIG in TSS, literature suggests MIS should globally be treated with immune-modifying agents, first-line glucocorticoids, and IVIG to reverse the inflammatory response. Glucocorticoids are strongly recommended for patients with KD with persistent fever after IVIG or coronary artery dilatation. In addition, current reports report that the combination of steroid therapy and IVIG may be more effective for symptom relief as compared to IVIG monotherapy in KD [11]. Therefore, the value of glucocorticoids as first-line treatment for hyperinflammatory syndromes remains undisputable, although the role of biologicals remains unclear to date. Although the exact effectiveness on long-term remains unclear, patients in limited case series show promising results [4, 11–16]. The proposed treatment guideline for patients diagnosed with MIS associated with SARS-CoV-2, according to The American College of Rheumatology, consists of the following:

1. IVIG
  The recommended dose of IVIG for patients with KD-like characteristics is the same as is used for KD, 2 g/kg (up to 70–80 g) body weight over a period of at least 8 to 12 h [11]. In some cases of no or inadequate response, the administration of a second dose of IVIG can be considered.
2. Glucocorticoids
  In severe cases with cardiac involvement, - TSS- or HLH-like course of the disease, high doses of glucocorticoids should be considered in combination with 1. For the recommended dose of glucocorticoids, methylprednisolone during early life-threatening stage a regimen of 1 mg/kg body weight daily, or in more severe cases based on clinical features and laboratory findings metylprednisolone 30 mg/kg pulse therapy once daily during 1 to 3 days, and in cases with secondary HLH or central nerve system involvement dexamethasone 10 mg/m^2^ once daily seems reasonable. When the disease has reached complete remission and the patient has improved clinically to be dismissed from hospital, the oral dose of prednisolone can be reduced over a period of weeks to minimize the risk of relapse. The supporting proof for using immune-modifying therapy is from previous case series, describing similar patient populations in same health conditions, like KD, HLH, and TSS. In these case series, 75% of the cases were treated alike with IVIG, who showed clinical and cardiac recovery after treatment [4, 12–15]. In other limited case series, approximately 55% of the patients were treated with glucocorticoids in different doses. Prior to administrating IVIG in these patients, it is indispensable to obtain blood for blood cultures in analysis of possible pathogens and serologic SARS-CoV-2 test.
3. Biologicals
  In cases with uncontrolled expanding disease activity, severe secondary HLH or shock by cardiac involvement despite the started therapeutic approach according to steps 1 and 2, the biological Anakinra (interleukin-1 receptor antagonist), attractive due to its safety profile and short half-life, is advised.

In our case, we added IVIG with high doses of aspirin to the therapy due to refractory shock and concerns of cardiac involvement in the later stage. Within 48 h after the onset of this therapy, his clinical condition recovered considerably, and cardiac left ventricular function has partially been restored up to 47%.

The risk of a relapse of and long-term complications from MIS after ceasing of the glucocorticoids, whether or not luxated by another pathogen, remains unknown. Complementary data are not available yet. Physicians should be aware of MIS during the first six weeks after an infection with SARS-CoV-2, so targeted therapy may promptly be initiated.

## Conclusions

SARS-CoV-2-associated MIS is a rare emerging clinical entity and has clinical features similar to several overlapping hyperinflammatory syndromes. Mainly children are affected several weeks after infection with SARS-CoV-2. Despite the similarities between MIS and other hyperinflammatory syndromes, MIS typically presents several weeks after SARS-CoV2 infection. Timely treatment with glucocorticoids, IVIG, and high doses of aspirin for those patients who are suspected for MIS should usually be sufficient to treat the inflammatory state. Further research is needed to clarify the aspects in the pathogenesis of MIS in young adult patients to improve effective targeted intervention.

## Data Availability

The data that support the findings of this study are available from the patient record system “HIX” at the Jeroen Bosch Hospital in The Netherlands, but restrictions apply to the availability of these data, which were used under license for the current case report, and so are not publicly available. Data are however available from the authors upon reasonable request and with permission of patient.
